# Learning from 30 years of systems thinking in healthcare

**DOI:** 10.3389/frhs.2026.1795979

**Published:** 2026-09-16

**Authors:** Christian von Plessen, Paul Batalden, Peter Lachman, Julie K. Johnson

**Affiliations:** 1 University Center for Primary Care and Public Health, University of Lausanne, Lausanne, Switzerland; 2 School of Health and Welfare, Jönköping University, Jönköping, Sweden; 3 Patient Safety Movement Foundation, Irvine, CA, United States; 4 Department of Surgery, University of North Carolina at Chapel Hill, Chapel Hill, NC, United States

**Keywords:** coproduction, healthcare quality and safety, product dominant logic, service dominant logic, systems thinking

## Abstract

Systems thinking is a foundational discipline for the quality improvement practitioner. A 1997 publication on building knowledge of healthcare as a system created a bridge between system thinking and quality management, summarized the thinking about systems thinking and quality improvement at that time, and created a 10-step exercise to guide participants in applying systems thinking in their own healthcare settings. During the past 30 years, our understanding of how systems thinking can be applied in healthcare has evolved. We now recognize several limitations to our previous thinking, in particular, the need to distinguish between product logic and service logic. Products are particularly well-suited for quality improvement initiatives that address standardization of linear processes while services are not. Further, we need to consider the role of coproduction for the person seeking care and the relationship and communication with the clinical team. Finally, we must design the system to include care that transcends organizational boundaries. In essence, we need to apply systems thinking to care that is non-linear, involves multiple organizations, and importantly, is coproduced with the patient. We propose a revised and updated systems thinking exercise to help healthcare leaders and patients to apply this discipline in their daily practice.

## 1 Introduction: from Deming to coproduction

The purpose of this paper is to plant a seed.

Deming taught systems thinking as one of four core disciplines of quality improvement (QI) (1). His ideas were instrumental in driving the quality revolution in manufacturing. While the concept of systems thinking was inspiring and conceptually compelling, learning how to be a systems thinker or, as Deming suggested, to “appreciate a system” has been a significant and prevailing challenge. In other words, it's easier said than done.

In 1997, we published an article on how to build knowledge of healthcare as a system. We wanted to build a bridge between system thinking and quality management and offer a practical approach to designing and improving healthcare (2). As part of the publication, we created an annotated 10 step facilitators' guide for those who wanted to try the approach in their own setting.

As we have continued using the exercise in our own organizations with health professionals and students, our understanding of systems thinking and how it can be mastered has deepened and evolved. Notably, with the growing complexity of healthcare and coordination problems across institutions, we have seen an increased need for individual and team practice of systems thinking within and across institutions (3). Also, we have understood that systems thinking, as propounded by Deming, is useful in some but not all areas of healthcare. His focus was on improving manufacturing and production and indeed, the application of Deming's ideas to healthcare has been successful for institutional care processes that are linear and benefit from standardization, for example, cataract surgery or knee replacement. Yet, their application to care for complex patients with chronic conditions or mental health problems has been less successful. Such care is non-linear, it involves multiple disciplines, often working across multiple institutions, and importantly, it is produced with the patient, not done to the patient (4).

The distinction between product and service logic differentiates between the two types of care. In his book The Service Economy, Fuchs notes “*Considerable rethinking of economic concepts may be required … one problem arises because the consumer frequently plays an important role in the production of services, but not in the production of goods.”* (5). Fuchs explained that the making of a service always requires two parties, although we usually only measure the productivity of one of the parties.

An example might be helpful in illustrating the difference between service and product logic in healthcare: Mr. Abernathy is working in his garden when he complains to his family of chest pain. His wife calls an ambulance. He arrives in the ED with a cardiac arrest and requires immediate resuscitation. At that point in the care process, the resuscitation is the *product*. It is “done to” Mr Abernathy by the team of healthcare professionals. Simultaneously, accompanying the family members in the waiting room belongs to the service logic. Reassurance, hope, comfort in a difficult situation are created *with* the family members and not *for* them. In the weeks and months following successful resuscitation, cardiac rehabilitation and its outcomes are also coproduced with Mr. Abernathy and his family.

Applying product logic to service–and vice versa–is misguided. Yet, that is exactly what has happened in healthcare. Partly driven by the QI and the patient safety movement, activities that would have benefited from QI based on service logic were treated as products. This confusion has contributed to the perception of healthcare organizations as a factory and the delivery of healthcare as a production line. Persistent quality problems, the questioning of the utility of QI, negative perceptions of healthcare, and professional burn-out are some of the consequences of that confusion (6, 7).

Thus, we believe our 1997 paper, while still valid in its practical and applied approach to building knowledge of healthcare as a system, is overdue for a revision. It must consider coproduced healthcare service at the level of the patient and healthcare professional, it must include healthcare that transcends institutional boundaries, and we must update the systems thinking exercise to help healthcare leaders and patients apply this discipline in their daily practice.

We first provide a brief overview of the original publication, discuss the gaps that emerged as our thinking has evolved, and review the additional concepts that bridge the gaps to how we currently think about health and healthcare as a system. We then present an update of the 10-step guide for the system exercise with new questions. With these changes we hope the reader will continue the inspiring exercise of “formulating the mess” of any system, as R Ackoff proposed (8).

## 2 Summary of Building Knowledge of Healthcare as a System

The 1997 article introduced systems thinking to healthcare improvement and proposed a 10-step exercise designed to help healthcare leaders understand and improve healthcare as an interdependent system, rather than a mere collection of entities. Our approach aimed to reduce waste and improve patient care by fostering systems thinking through a guided approach of inquiry, reflection, and discussion.

The definition of a system was an important component of our exercise. We built on the work of prominent systems thinkers (9, 10), and proposed W. Edwards Deming's model of production as a system (Figure 1) as the foundation for the 10-step exercise. The key components of Deming's system model show “Suppliers of materials and equipment”, the process of “Production, Assembly, Inspection, Distribution”, and “Consumers”.

**Figure 1:**
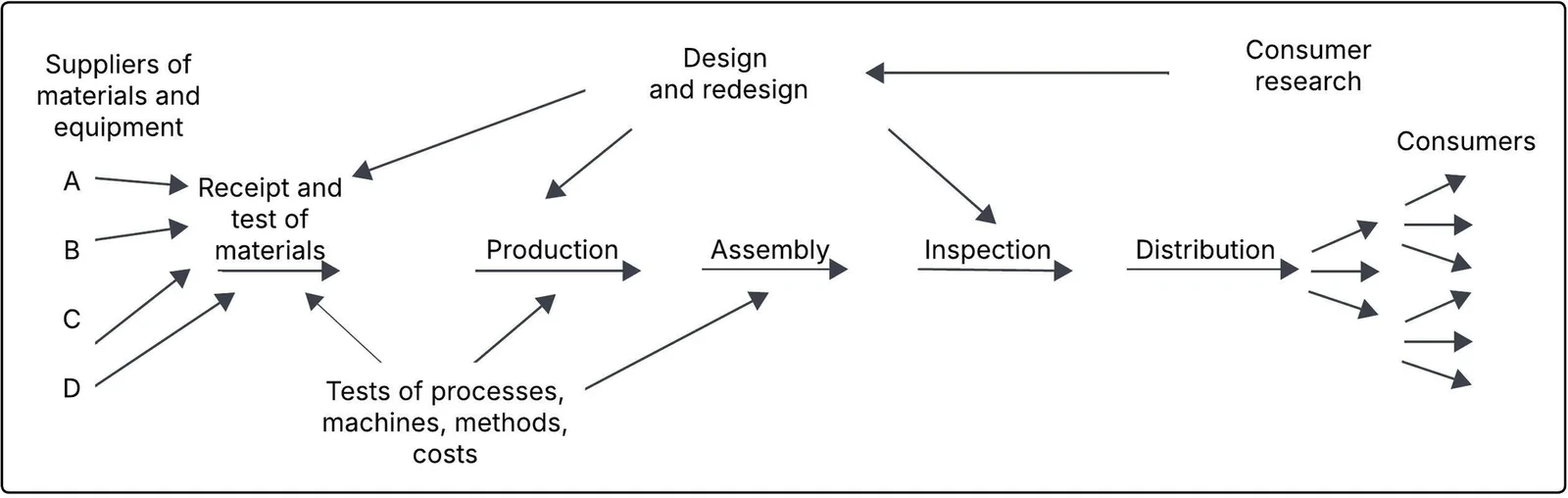
Deming's model of production viewed as a system [Deming (1)].

Deming viewed the consumer as part of the system of production, in the sense of a potential receiver of the product. For Deming, the consumer was “*the final judge of quality*”, who executes this judgement by either buying or not buying the product. We adapted Deming's model to make it understandable for healthcare improvement by collapsing Deming's steps of the process, by adding a “Vision”, a “Plan to improve” and by changing “Consumer research” to “Community need” (Figure 2).

**Figure 2:**
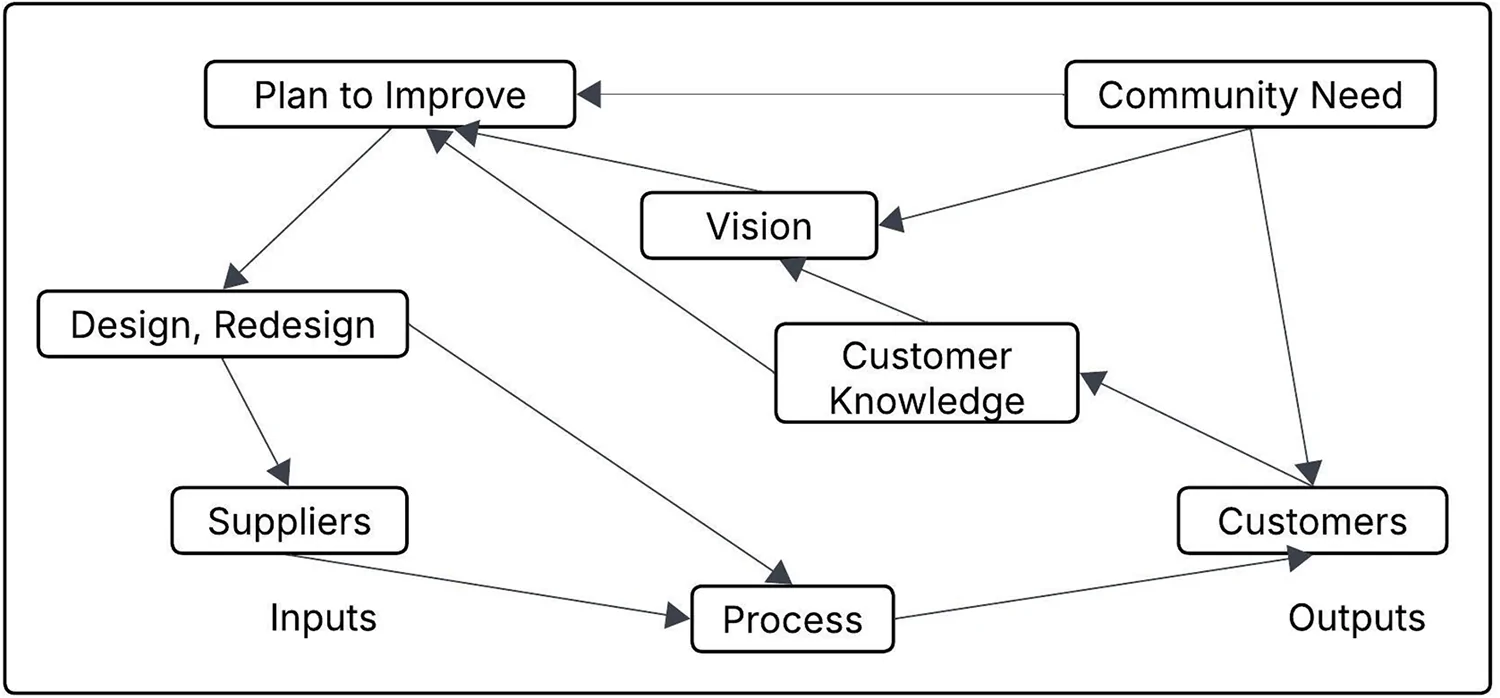
A system capable of continual improvement [Batalden and Mohr (2)].

Our aim was to help managers and clinicians recognize the system they work within and understand how different components within the system interact. By asking them why they make what they make, how they make it and how they can improve what they make, we wanted to focus their attention to practical applications that would lead to improved processes and outcomes. Realizing systems thinking requires practice and careful facilitation, we designed a 10-step exercise to guide participants toward an “*appreciation of a system*” (1) with a particular focus on the system they want to improve. In this sense it was inspired by the tradition of “action research” or “insider action research” (11) as well as our research into understanding the functioning of clinical microsystems (12–17). It helped participants see their own daily work (what they make) within their system. We applied Deming's production “lens” to healthcare and encouraged and taught this method as a way for institutions to improve their processes to provide better patient care.

While this approach represented our thinking about improving the quality of care in the late 1990s, today, three main evolutions challenge it:

1. The role of patients and the understanding of their contributions to making health have changed.
2. The industrialization and the application of product dominant logic to healthcare threatens the therapeutic relationship and limits the effect of improvement efforts.
3. Organizational quality improvement is inefficient in solving quality and safety problems across healthcare organizations.

In the next section, we present the evolution of our thinking with regards to these three aspects, introduce key concepts, and propose a revised systems thinking exercise.

## 3 How our understanding limited Building Knowledge of Healthcare as a System

### 3.1 The role of patients and the understanding of their contributions to making health have changed

L.J. Henderson stated in an address to the Harvard Medical Colloquium in 1934 “*A physician and a patient make up a social system*” (18). His insight was visionary then. Today, nearly 100 years later, we have a better understanding of what he meant, and we cannot ignore how improved access to medical information has contributed to diminished asymmetry between doctors and patients and the democratization of healthcare. Further examples of challenges to health professional paternalism include the 2010 Montreal Model which recognized the patient's experiential knowledge gained from living with a disease as complementary to the healthcare professional's scientific knowledge (19). Shared decision making, despite lagging clinical implementation, is increasingly considered a standard in many practice guidelines. Finally with the increase in chronic diseases, it becomes clear that health is “made” by persons with diseases with help from persons with professional knowledge and that patient autonomy and agency in this endeavour are key. Henderson's observation in practice means to reconsider roles, relationships and interactions between patients and professionals at all system levels and facilitate “making together” what contributes to health.

Dumez and L’Esperance (20) describe the six types of knowledge that patients may bring to the patient—health professional relationship:

1. Embodied knowledge is sensory and grounded in bodily experience, anchored in physical sensations (pain, relief, discomfort, level of energy, etc.) and perceptions of the body and mind.
2. Monitoring knowledge is the capacity to identify with acuity physical, mental, and/or emotional signs either through formal tracking or self-awareness.
3. Navigation knowledge comes from the repeated use of health care and services, from the repeated experience of institutional access and barriers to health care and services, and from searching and finding resources available within and outside the healthcare network.
4. Medical knowledge is the derivative of two dynamics: (1) meeting with medical practitioners and (2) seeking information about the physiological causes and mechanisms of their illness, their symptoms, the options and types of treatments, the side effects and benefits of such treatments, the drug interactions, etc.
5. Relational knowledge is a set of explicit and situated knowledge that enables patients to know who to turn to within their community to access the care they need, to help them with disease management and support the achievement of their lifegoals.
6. Cultural knowledge includes the norms, values, symbols, constructions of reality, and worldviews that influence the experience of life and illness.

### 3.2 The industrialization and the application of product dominant logic to healthcare threatens the therapeutic relationship and limits the effect of improvement efforts

Relational and communication difficulties are among the most common patient complaints today while, at the same time, a growing number of professionals feel alienated from their work (21). Patients see themselves reduced to parts being fixed in repair shops that apply standardized procedures. Healthcare professionals in hospitals, practices, and home care increasingly feel like factory workers doing repetitive and meaningless work. In the USA, private equity funds increasingly invest in what commonly is called the *healthcare industry* and returns on these investments are expected through increasing production of healthcare actions to the detriment of quality (22–25). Despite major successes in areas such as anesthesia that have benefitted from standardization and other QI approaches inspired by the production industry, QI is often considered a contributor to this alienation through measurement, benchmarking and standardization (26, 27). These approaches are often perceived as useless bureaucracy distancing health professionals from patients. The logic of product–and most mechanisms of remuneration–underlie this development. Today we must question the broad application of the product logic to virtually all areas of healthcare, even to those who might benefit from applying a logic of service to create value for patients and society (6, 28).

### 3.3 Organizational quality improvement is inefficient in solving quality and safety problems across healthcare organizations

Albeit some indisputable successes, QI seems to be in a crisis, and the return on investment of QI efforts has been questioned (29). Frequently heard critiques include QI has been too hospital centric, effects at scale are lacking and it is too costly. It is true a lot of QI is done at institutional, often project level, thus not achieving effects at scale (7) and smaller institutions often lack the means and competencies for QI. Yet, many quality problems arise at the boundaries and interfaces between institutions and it comes as no surprise “coordination” has been added to the original six QI dimensions of the IOM report (30). Also, the growing awareness of the contribution of healthcare to ecological problems increases the visibility of system issues (31). Finally, despite long-standing recognition that most health problems are managed and resolved in primary care, this has not been accompanied by an equivalent level of engagement in QI within the sector. Thus, when we today intend to appreciate a system capable of improvement, we must broaden our scope and include patients and professionals in- and outside dominating institutions.

## 4 Revision of Building Knowledge of Healthcare as a System

We propose to modify our approach as presented in 1997 to:

1. include patients in preparing and carrying through systems thinking exercises to acknowledge coproduction by patients and healthcare professionals.
2. identify, distinguish and differently treat processes that correspond to a product or a service logic (See the callout for “process” in Figure 3).
3. challenge the institutional focus of the original exercise and explore the boundaries acknowledging the embedded nature of healthcare organizations and their interdependence.
4. invest in education of the coproduction of systems thinking to promote this approach.

**Figure 3:**
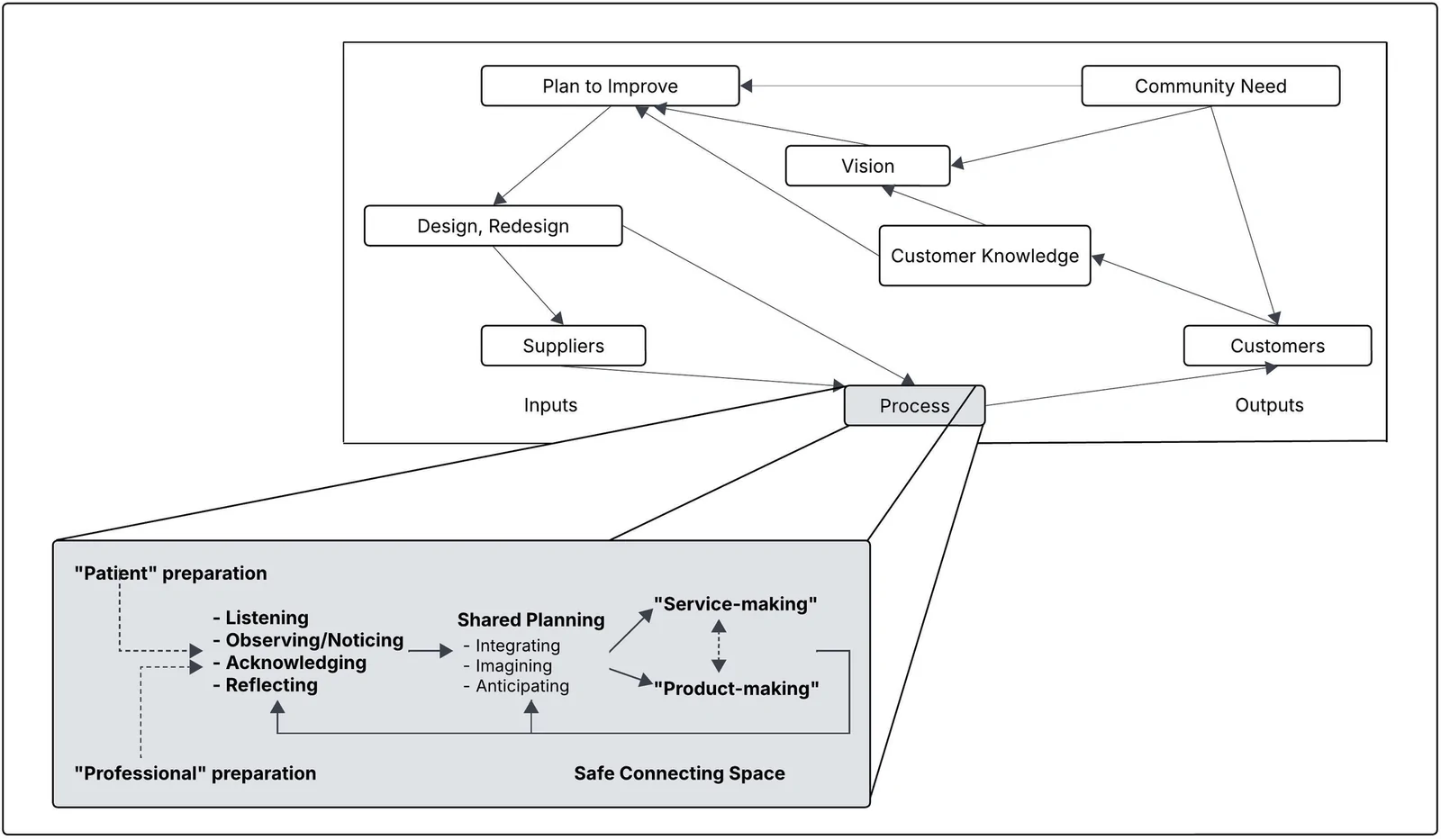
Revised view of a system capable of continual improvement.

To reflect these aspects, we have modified our 1997 illustration of a system capable of continual improvement that was based on Deming's Model. (See Figure 3).

In addition, we have modified the systems thinking exercise (Figure 4). It is now presented as a flow diagram that invites the user to identify if the exercise is about the creation of a service or a product (Step 1: Service or product). We also invite the user to engage in pre-exercise review and planning and post-exercise reflection. Otherwise, we have kept the structure of the original exercise (Steps 2–10) and adapted the outputs of steps 8–10 and added questions and comments according to the type of value creation model, service or product. Table 1 provides guidance to facilitators of the exercise by listing the steps and providing definitions and suggestions for facilitating the exercise. To further support those who would like more guidance on the exercise, a case study is provided as a Supplementary File.

**Figure 4:**
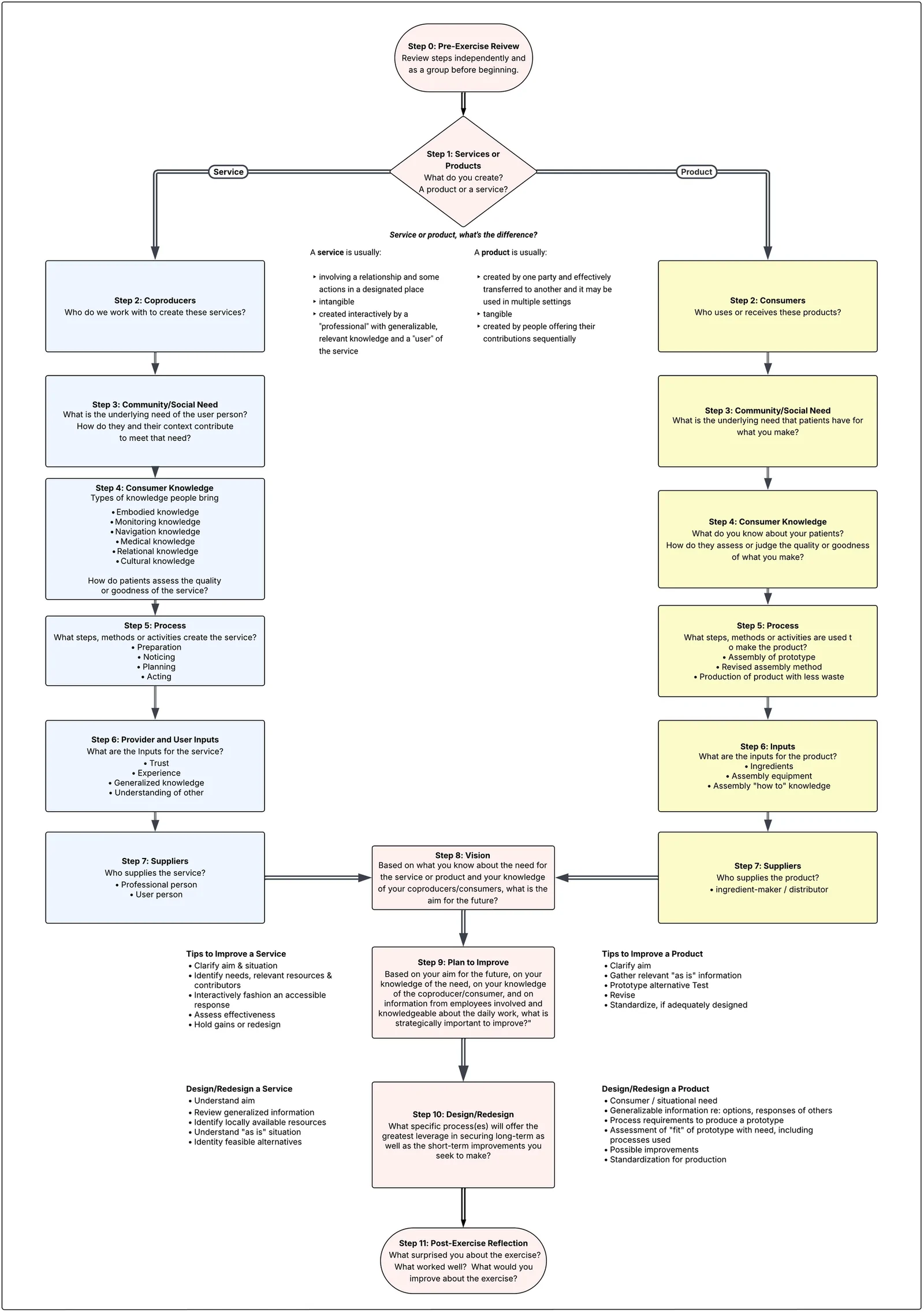
Building knowledge of a healthcare as a system.

**Table 1:** Guide for facilitators with explanations of terms and constructs.

Step and construct	Construct definition and suggestions for facilitators
Step 0: Pre-Exercise Review	At the start of the session, ask participants to review the exercise independently and note their initial thoughts for each step. As a group, review the exercise and explore which steps participants think will be the easiest for the group and which will be most challenging. Make note of these comments without attempting to resolve or debate the issues.
Step 1: Service or Product	The first activity is to define what you make or create a product or a service or a combination of both. This may be a new way of thinking for the participants.
	Service(s) are usually intangible and involve a relationship and some actions. Importantly, they are created interactively by a "professional" with generalizable, relevant knowledge and a "user". A decision about surgery might serve as an example.
	Product(s) are usually created by one party and effectively transferred to another. They are often tangible or predefined processes, for example an algorithm for resuscitation.
	Note that categories of product and service are not always black and white.
	The exercise invites participants to explore the differences between service and product and to consider how they can provide a highly reliable product at the same time providing a service.
Step 2: Coproducers or Consumers	Who uses or receives these services or products? Who might benefit? Start with external coproducers or consumers. List those most often encountered to identify who benefits from the services and products you make. The list may include some combination of patients, families, physicians, clinical staff, employers, and payers.
Step 3: Community/Social Need	What is the underlying need for what you make or create? Sometimes it is helpful to take each coproducer or consumer listed in Step 2 and ask about their need for what you make. Remember that the focus is on “need” not what you or anyone else does to meet that need. Keep digging until you get to a level that permits some flexibility in the design of new services. Are there other, related needs that are not being met?
Step 4: Consumer Knowledge	The two questions to answer here are, “How do consumers assess or judge what you make?” and “What drives their assessment?”
	It helps to start with a single consumer. Identify the service or product you create, and ask how that specific consumer assesses quality or "goodness" about that product or service. The typology of patient knowledge by Dumez and L’Esperance might be helpful here: health professional relationship to include, embodied knowledge, monitoring knowledge, navigation knowledge, medical knowledge, relational knowledge, and cultural knowledge.
	Then focus on what might prompt each consumer (or consumer group) to assess and judge quality that way. What are their reasons for that quality characteristic? If you are using a whiteboard or flip chart pages to document responses, connect those "prompts" to the measures of quality with an arrow from the prompt to the measure. These prompts can be thought of as "drivers" of the measures of quality and goodness.
Step 5: Process	"What activities are needed to make the service or product?" In creating a service, we imagine several steps that are part of the process of creating a service, namely, preparation, noticing, planning, and acting. These service creating steps are distinct from product creating steps of assembly of prototype, revised assembly method, production of product with less waste.
	It helps to think of a typical scenario for the consumers of your services and products.
Step 6: Provider and User Inputs	Here, the question is, "What comes into your process and is changed by the regular actions of the process to create the services or products?" The answer to this question is to acknowledge the entering needs, skills, materials or goods that regularly enter your system and form the beginning point to your work.
	Remember that inputs come from internal as well as external sources and include human, financial, material and information inputs to your processes.
	For services, inputs include trust, experience, generalized knowledge, understanding of the other.
	Be aware that the supplier list is next. Do not become confused between the two. Suppliers supply inputs. The input list is for what suppliers supply. For example, employees are sometimes listed as inputs, but actually, the inputs are employee skills and knowledge, while employees are the suppliers.
Step 7: Suppliers	Who supplies the inputs for the services and products you create? For each input in step 6, list the supplier(s) of patients, materials, manpower. List suppliers of "patient need," "physician skills", etc.
	Sometimes the same person or department can be supplier and consumer of the process. If so, they supply one thing and receive another, presumably your process has added value to what they have supplied.
	Remember that the concept of supplier applies to inside and outside your organization. For example, when a doctor orders an Xray the doctor is both a supplier of a diagnosis for the person receiving care and a consumer for the Radiology department which is the supplier of Xrays.
Step 8: Vision	Based on what you know about the need for the service or product and your knowledge of your coproducers and consumers/patients, what is the vision for the future? What is the interaction between product and service to give value to the consumer?
Step 9: Plan to Improve	Based on your aim for the future, on your knowledge of the need, on your knowledge of the consumer, and on information from employees involved and knowledgeable about the daily work, what is strategically important to improve?
Step 10: Design/Redesign	What specific process(es) will offer the greatest leverage in securing long-term as well as short-term improvements you seek to achieve? How can you move between product and service delivery?
Step 11: Post-Exercise Reflection	Before adjourning, ask participants to reflect on the exercise for a couple of minutes on their own. Ask them to be curious about their own context. You can use these questions as prompts for reflection: What happened as we did the exercise? What surprised you about the exercise? What worked well? What would you improve about the exercise? What should we do next? After participants have 5 min to answer these questions on their own, ask them to share their reflections with the group.

Building Knowledge of Healthcare of a System is an exercise intended for those who want to learn about the system they work within. It is possible to do the exercise at several system levels (macro, meso, micro), and it is important to be clear about the level you are addressing, i.e., a region, a hospital, a department, a community health center, etc. Starting with the level of the organization which everyone has in common is helpful. At that level people have common knowledge. A whiteboard or flip chart pages would be useful for documenting the responses to the steps.

Finally, we are developing learning materials and tools to support health professionals to learn how to coproduce (32). For introductory materials the reader can refer to a series of podcasts exploring different aspects of coproduction of healthcare service (available for free on the International Coproduction of Health Network (ICoHN): https://www.coproducehealth.com/podcast/, a special issue about coproduction in the International Journal for Quality in Health Care (33), and numerous courses, for example, the coproduction track and learning journeys at the annual conferences of the International Society for Quality in Health Care. ICoHN, a loosely coupled interdisciplinary network, is a hub for many of these activities (34). ICoHN recently launched a series of learning events where clinicians and patients share their experiences with applying a 9 dimensional template for practical coproduction (32). These virtual international learning events have brought together colleagues from Asia, Africa, Europe, Central and South America, Canada, and the US. The free events are open for anyone who could benefit from trying out coproduction in clinical settings (please contact the authors for further information).

## 5 Discussion

Quality improvers need systems thinking skills to improve the contribution of healthcare to health. Appreciating systems requires continuous inquiry and learning to maximise value for patients and professionals and to avoid system suboptimisation. It also requires continuous adaptations to the development of healthcare, technology, and roles of patients and professionals as well as our understanding of the limitations and strengths of different QI efforts.

Much has changed since the introduction of systems thinking to healthcare QI in the late 1990s, notably the understanding that a major part of value creation in healthcare follows a service logic and that health professionals and patients coproduce these services in increasingly complex healthcare systems.

Distinguishing three periods of healthcare quality development can help understand the introduction of new paradigms and methods (Table 2), such as coproduction, to healthcare (35, 36). Quality 1.0 corresponds to the early efforts of quality assessment and assurance by Florence Nightingale, Ernest Codman and others in the late 19th and early 20th century that led to the establishment of standards and norms and applied methods such as accreditation. Quality 2.0 marks the transfer of industrial QI to healthcare in the late 1990's (when we published our paper on systems thinking) inspired by its successes in production industries such as in car making in the early 1980s. Finally, during Quality 3.0, beginning around 2010 and accelerated by the pandemic, it became clear that the product dominant logic of the production industry cannot be universally applied to healthcare. A service dominant logic seemed more suitable for improving the contribution of healthcare to health because health, as any service is, is coproduced by patients and professionals (4).

**Table 2:** Evolution of improvement work.

Quality 1.0	Quality 2.0	Quality 3.0
Thresholds	Organization-wide systems	Coproduction of health
What is the threshold of a good hospital, clinic, setting?	What is the best system for a situation/disease/condition?	How might we improve the value of contribution that “service” makes to health?
Accreditation	System/process	Lived reality of the patient/family/ professional
Standards	Variation & “statistical thinking”	Ownership of “health”/ “welfare”
Audits/inspections	Learning from testing change	Service & product
“Be at least this good…” (floor)	“Be as good as possible” (ceiling)	Service coproduction

The proposed changes will require new competencies, organisational adaptations and investments in terms of time and money.

### 5.1 Include patients in preparing and carrying through systems thinking exercises

Guidance about patient involvement is available, for example, from the Institute for patient and family centered care (http://www.ipfcc.org) (37), but operationalization of coproduction at the organizational level still varies widely. Key questions to address include: which patient characteristics should guide selection, how to identify and recruit participants, how to ensure diversity of experience and background, whether and how to remunerate contributors, and how to adapt work processes to different levels of prior knowledge and health literacy (38).

In addition, issues of representativeness and the legitimacy of the patient voices included must be carefully considered. Also, from a practical perspective, improvement initiatives should explicitly define the extent and modalities of patient involvement. The project's objectives and the resources available to support meaningful engagement will inevitably shape these decisions.

While conceptually compelling, integrating patients and professionals as full partners in systems thinking exercises requires substantial shifts in entrenched organizational cultures, professional hierarchies, and professional identity norms. The inclusion of patients on improvement teams inevitably raises questions of power asymmetry between patient and professional roles, and the risk of tokenistic participation remains a well-documented concern (39, 40). Ensuring authentic and productive engagement may therefore require preparation and accompaniment for team members and patient partners, including the development of mechanisms to address—or, when necessary, to conclude—collaborations that prove unproductive or unsustainable (41).

### 5.2 Challenge the organizational focus of the original exercise by exploring boundaries

Individual health needs transcend traditional care levels and organizational boundaries. Yet health systems remain structured in sectors and institutions. Resource allocation and financing follow the same logic and contribute to maintaining the silos.

This usually becomes more visible for improvement and management teams during system exercises and the participation of patients will increase this visibility. Their system of making health does not or only partially or periodically match the institutional healthcare system. Paradoxically, this can lead to an impasse because the improvement team and participating patients face barriers that they cannot overcome or only with great investment of effort and time.

So how can this be avoided without falling into the trap of disregarding the embedded nature of healthcare organizations? First, improvement teams need to appreciate that operating systems of health and care service exist in wider systems and/or environments with which they interact (42). Clarifying and communicating clearly the scope and actionable aspects of a project as well as identifying escalating mechanisms for issues beyond scope can be helpful. Also looking for connectors in terms of boundary spanning roles, data flows, and ways of collaboration avoid loss of momentum and frustration.

### 5.3 Seek to identify, distinguish and differently treat processes that correspond to a product dominant or a service dominant logic

The language of product and service can be confusing and neither healthcare professionals nor patients are familiar with it. The following questions might be helpful in identifying product and service in clinical processes:

- Is the activity done *to/for* the person or *with* the person?
- Is the value created *before* or *during* the activity?
- Is the result of the activity a thing, a skill-based, standardized action or is it immaterial, an interactive process?

Products are made for someone, they are developed or made before they are used and they are often things, algorithms or standardized processes. Many skill-based healthcare activities, such as cardiac resuscitation in our example above, fall under this category. Service on the other hand is done with the person, i.e., coproduced, the value is created in the moment of the interaction between the professional and the patient and it is always immaterial (Figure 3).

It can be difficult to distinguish the two types of value creation. Care processes often include both in sequence or in parallel. Grey zones are common as captured by Vargo and Lusch's (45) use of the terms of “product-dominant and service-dominant” logics. Nevertheless, the distinction is important because applying service logic to product or vice versa is counterproductive and contributes to inefficient quality improvement.

### 5.4 Invest in training in the coproduction of systems thinking

The mastery of systems thinking and of the coproduction of systems thinking require knowledge and skills. Our approach combines learning about systems thinking and learning to use it in practice. The multimodal learning resources outlined in Table 3 include (articles, podcasts, videos) as well as tools for experimenting in practice (systems thinking exercise, Figure 4, coproduction template (32) and collective reflection (virtual learning sessions). We welcome improvers and patients to use these freely available resources in their explorations and practice tests.

**Table 3:** Learning resources for coproduction available from www.coproducehealth.com

Resources
Multimodal learning resources
Articles, podcasts, videos
Podcasts
Videos
Tools for experimenting in practice
Systems thinking exercise
Coproduction template
Collective reflection
Online learning sessions

We are aware that these propositions might seem like asking too much from already busy and desperate healthcare professionals. Some might feel that exploring their systems confronts them with an unsolvable crisis. We do not claim to have the solutions to this crisis, but the revised systems thinking exercise (Figure 4) is one place to start to connect to resources that can be used for improving the contribution of healthcare to health and thus address the roots of that crisis. The exercise will benefit from testing.

Appreciating systems in Deming's sense invites never ending learning about how processes can be improved. Learning how to coproduce healthcare services invites action research into our own organizations, their interdependencies with other organizations, with patients and their families, and how they are embedded in larger systems. Indeed, they are a roadmap for action research into your organizations, units, practices (43).

Our intention is to plant a seed that we hope will germinate (44) into new ways to improve the quality of healthcare.

## 6 Conclusion

After this revision, we are convinced that systems thinking, with the proposed extensions of coproducing with patients, transcending the institutional perspective, distinguishing product and service, and creating learning opportunities for coproduction, remains a foundational discipline for the quality improver to master.

Our proposed modifications of the practice of systems thinking for healthcare QI are based on the practical experiences of the authors and have been discussed among us. We do not claim that they will solve the pressing problems of healthcare improvement today. Rather, we consider them an invitation to reframe these challenges and to include all relevant actors in addressing them. We encourage practitioners to test the exercise in their work and we would very much welcome feedback and to hear about your experiences.

## Data Availability

Publicly available datasets were analyzed in this study. This data can be found here: N/A.

## Glossary

Acknowledging: Recognizing, accepting, or showing appreciation for a person's thoughts, feelings, contributions, or experiences.
Anticipating: Thinking ahead to predict possible needs, challenges, or outcomes and preparing for them.
Community need: Perceived requirement of people to live healthy, safe, and productive lives.
Consumers: The consumer could be a person receiving care or another healthcare provider receiving a product or service, e.g., a lab result. They benefit from a product or service, whether or not they purchased it.
Customers: Individuals or organizations that purchase or receive a product or service.
Customer knowledge: Understanding customers' needs, preferences, expectations, and experiences to provide better products or services.
Design, redesign: Design is creating a new product, service, or process. Redesign is improving or changing an existing one to make it more effective.
Imagining: Using creativity to think of new ideas, possibilities, or solutions.
Integrating: Combining different ideas, information, skills, or resources into a unified approach.
Listening: Paying close attention to what another person is saying to understand their message, feelings, and motivations.
Observing/Noticing: Carefully watching or recognizing details, changes, or behaviors.
Patient preparation: The physical, emotional, and educational steps taken to get a person ready for a procedure, treatment, or healthcare experience.
Plan to improve: A step-by-step strategy for making a process, service, or outcome better.
Process: A series of connected steps or actions taken to achieve a specific result, or to create services and products. Service creating steps include preparing, noticing, planning, and acting. Product creating steps include assembly of prototype, revised assembly of product, production with less waste.
Product: Product(s) are usually created by one party and effectively transferred to another. They are often tangible or predefined processes.
Product making: The process of designing, producing, and improving physical goods to meet customer needs.
Professional preparation: The education, training, skills, and planning needed for a professional to perform their role effectively and safely. Service Service(s) are usually intangible and involve a relationship and some actions.
Reflecting: Thinking carefully about experiences or actions to learn from them and improve future performance.
Safe connecting space: An environment where people feel physically and emotionally safe to communicate openly, share ideas, ask questions, and collaborate without fear of judgment or harm.
Service making: The process of creating, improving, and delivering services that meet the needs of customers or patients.
Shared planning: Working together with others, such as patients, families, or team members, to make decisions and develop a plan.
Social need: Perceived requirement by people for relationships, acceptance by others, participation in society, contributing to well-being. Suppliers, People or organizations that provide the materials, products, or services needed by another organization.
Vision: A clear picture or goal of what you want to achieve in the future.
