# Supplementary material for: Learning from 30 years of systems thinking in healthcare
Source: Front Health Serv. 2026 Sep 16;6:1795979. doi: 10.3389/frhs.2026.1795979 (PMC13624103; doi:10.3389/frhs.2026.1795979)
Supplement: Supplementary file 1 [file Datasheet1.pdf]

Building Knowledge of Healthcare as a System:

A case study the Comprehensive Stroke Care Center at Good Samaritan

Time is of the essence in the management of a person who presents to the hospital with an acute ischaemic stroke. As each minute that passes, the brain loses 1.9 million neurons and 14 billion synapses.(1) The Comprehensive Stroke Care Center at Good Samaritan (referred to by the community as "Good Sam") has been recognized as a leader in stroke care since 2019 and has received multiple awards for their superior care. Good Sam has a multidisciplinary stroke care team with specialists in neurology, vascular surgery, emergency medicine, and physical medicine and rehabilitation. The stroke care team also includes training opportunities for residents and fellows and that is the nidus for our case study.

Dr. Craig completed her residency and is starting a one-year fellowship in stroke/vascular neurology at Good Sam. In addition to her clinical training, she signed up for a quality improvement project about improving door to needle time of the stroke pathway. Dr. Craig's idea is to build on her previous work in coproduction where she had applied the concept of service and product dominant logic.

She started by introducing the team to the concepts of service-dominant logic and product-dominant logic. As she explained at one of her first team meetings, "*To me, the concepts of service and product are clearly defined when we consider the person's journey which starts at home when the ambulance is called.*" She continued, "*I can take the team through an exercise that will help us think about stroke care at Good Sam as a system*." Optimistic about new ways to improve how they work as a team, they set time to meet.

Next Thursday morning as the team sat down and got to work, Dr. Craig asked them to think about activities that follow a service logic and those that follow a product logic and what the distinction means for stroke care. Everyone took a few minutes to reflect individually on what the Comprehensive Stroke Care Center creates and then shared their ideas (Step 1).

Products the team identified were tangible outputs that support decision-making or treatment (blood tests, registration, triage outcome, CT scan, thrombolysis, thrombectomy, therapy assessments).

Services were interactions, decisions, care and caring activities, communication, coordination, support, and transport. There was an emphasis on supporting the family and kin. As the team talked, they realized that there are **combinations** of product and service dominant activities can occur in sequence, in parallel, in combination, but that they should not be confused.

Energized by their discussion about services and products, the team turned to Step 2 to identify the "coproducers" of the services and the "consumers" of the products**.** At this point in the exercise, the team had established that stroke care offers multiple services and products. Services are created with the service user or "coproducer". The consumers of the products might be the same people, but they are receiving something, not coproducing it. In stroke care, the products need to be ready to be in action without delay for the consumer – paramedical intervention, registration, triage, CT scan, then thrombectomy and or thrombolysis if indicated. This is followed by a swallowing assessment within 4 hours and then interventions like physiotherapy, occupational therapy and psychological assessments and support.

The team was confident they knew the community/social need (Step 3) for the services and products the stroke care team created. Good Sam's Comprehensive Stroke Care Center is part of a safety net hospital and is the only dedicated stroke care team in the region. When Good Sam started the stroke center in 2019, their community was experiencing mortality rates due to stroke roughly 40% higher than the nation's average. So, the community need and the resulting mission for Good Sam were clear, but thinking of service, they also realized that there might be needs that were not met by their current setup.

The team was less confident about what they knew about the consumers and the type of knowledge people bring and how they assess the value of the goods and services (Step 4). They actually have a lot of data that they collect to comply with accreditation standards for comprehensive stroke centers, but as they talked about the data they have and their benchmarked measures, they realized that they don't

know as much about the lived experience of the person who receives stroke care.(2) Or the people who help take care of them. Dr. Craig noted that this might be a good point to come back to when the team gets to Step 9, Plan to Improve.

Process (Step 5), inputs (Step 6), and suppliers (Step 7) were easier for the team to think about. In fact, much of their improvement work is grounded in process improvement methodology. Maintaining their accreditation as a Comprehensive Stroke Care Center requires them to meet certain standards that are related to the care process.

Dr. Craig led the team through a quick process mapping exercise to capture a high-level view of stroke care, from a patient perspective:

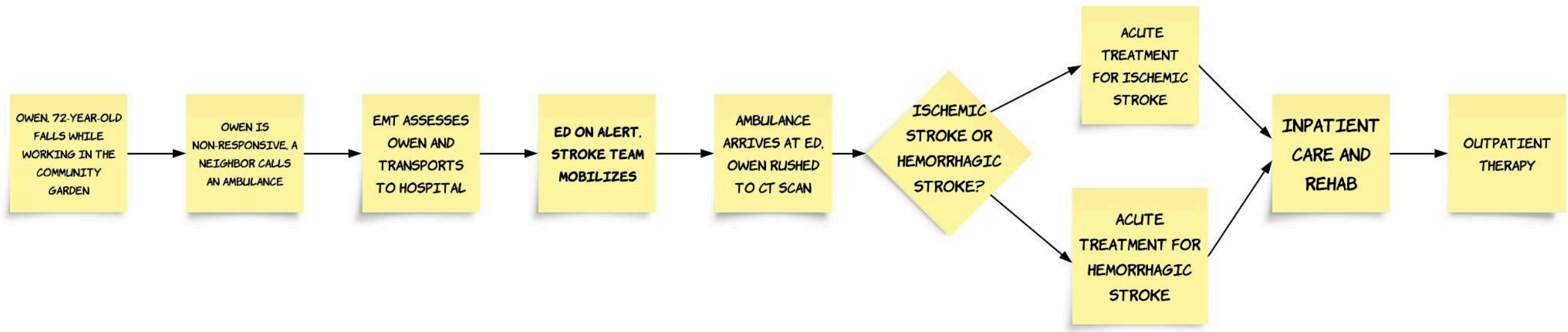

This high-level view prompted the team to go back to the list of products and services identified in Step 1 and create a more comprehensive list, shown in the following table that detailed each step in the process and included whether that step creates a service, product, or combination.

Acute Ischemic Stroke Process and Classification of Service and/or Product

Process Activity	Classification	Rationale
Stroke symptoms occur at home	Service Trigger	Initiates the care pathway.
Family calls ambulance	Service	Request for emergency response.
Ambulance dispatch and arrival	Service	Timely transport and emergency response.
Paramedical assessment, history taking, support of patient and family	Product + Service	History, observations, and blood tests are products; empathy, communication, and support are services.
Baseline blood tests	Product	Diagnostic information generated for decision-making.
Transport to hospital	Service	Delivery of emergency care and transport.
Registration in Emergency Department	Product	Administrative process producing patient record and entry into care system.

Triage	Product	Structured clinical assessment producing prioritisation and urgency classification.
CT Scan	Product	Diagnostic imaging result used for treatment decisions.
Family support and communication	Service	Emotional support and information sharing.
Clinical assessment and review of investigations	Product + Service	Interpretation of diagnostic products combined with clinical interaction.
Consent discussion with patient and family	Service	Shared decision-making and communication process.
Treatment decision (thrombolysis vs thrombectomy)	Product + Service	Clinical decision based on diagnostic products and collaborative discussion.
Thrombolysis	Product	Specific treatment intervention delivered.
Thrombectomy	Product	Specific procedural intervention delivered.
Peri-procedural support for patient and family	Service	Ongoing care, communication, and reassurance.
Post-stroke care planning	Product + Service	Care plan is a product; collaborative design and discussion are services.
Swallow assessment (Speech & Language Therapy)	Product (standalone) / Service (integrated MDT)	Assessment result is a product; integrated MDT care becomes a service.
Physiotherapy assessment and rehabilitation	Product (standalone) / Service (integrated MDT)	Assessment/treatment plans are products; coordinated rehabilitation is a service.
Occupational Therapy assessment	Product (standalone) / Service (integrated MDT)	Functional assessment is a product; coordinated care is a service.
Psychology assessment and support	Product (standalone) / Service (integrated MDT)	Assessment output is a product; ongoing support is a service.
Multidisciplinary Team (MDT) rehabilitation programme	Product + Service	Integration of multiple products into a coordinated rehabilitation service.
Ongoing recovery and discharge planning	Product + Service	Care plans, discharge documents, and follow-up arrangements combined with patient support.

The team agreed to stop after Step 7 and reconvene next week to tackle Steps 8, 9, and 10.

When the team came together to finish the exercise, Dr Craig asked, “Given everything we talked about and based on what you know about the need for stroke

care in our region and knowledge of our coproducers and consumers, what is our aim for the future?"

Jess, one of the nurses on 3 West spoke up, "I always liked our vision to provide unparalleled multidisciplinary stroke care, but now I'm wondering if we need to say something about coproducing stroke care with patients and families." Others in the room nodded, and Dr. Craig suggested that they create a smaller working group to tackle updating the Stroke Center's vision statement.

Dr. Craig brought everyone's attention to the last two steps in the exercise, Plan to Improve (Step 9) and Design/Redesign (Step 10). "Based on what we've talked about, what do you think is strategically important to improve?" Dr. Garcia, the Medical Director of Good Sam's Comprehensive Stroke Center, who had been uncharacteristically quiet during this exercise, said, "We have several QI projects underway based on our national benchmarks. We are working on reducing door to needle time, we want to implement a telestroke network for the region . . . but at this point, I think we need to spend more time understanding our "coproducers" as Dr. Craig calls them." Dr. Garcia paused and looked around the room. "Dr. Craig, I want you to lead this effort in teaching us about coproduction and, more importantly, how to coproduce stroke care."

"One more thing," Dr. Garcia added, "I see that there is a final question on the exercise diagram that asks, 'what surprised you about the exercise'. I'm curious to hear from everyone else, but I'm surprised that the distinction between service and product is so relevant to our work providing stroke care.

This represented a move from improving product delivery to one of including service in the service redesign.

Citations

1. Saver JL. Time is brain--quantified. Stroke. 2006 Jan;37(1):263-6. doi: 10.1161/01.STR.0000196957.55928.ab. Epub 2005 Dec 8. PMID: 16339467.
2. Wolf J, Niederhauser V, Marshburn D, Lavela S. Reexamining "Defining Patient Experience": The human experience in healthcare. Patient Experience Journal. 2021;8(1):16-29. doi: 10.35680/2372-0247.1594
